# Supplementary figures and images for: aTBP: A versatile tool for fish genotyping
Source: PLoS One. 2020 Aug 4;15(8):e0237111. doi: 10.1371/journal.pone.0237111 (PMC7402489; doi:10.1371/journal.pone.0237111)

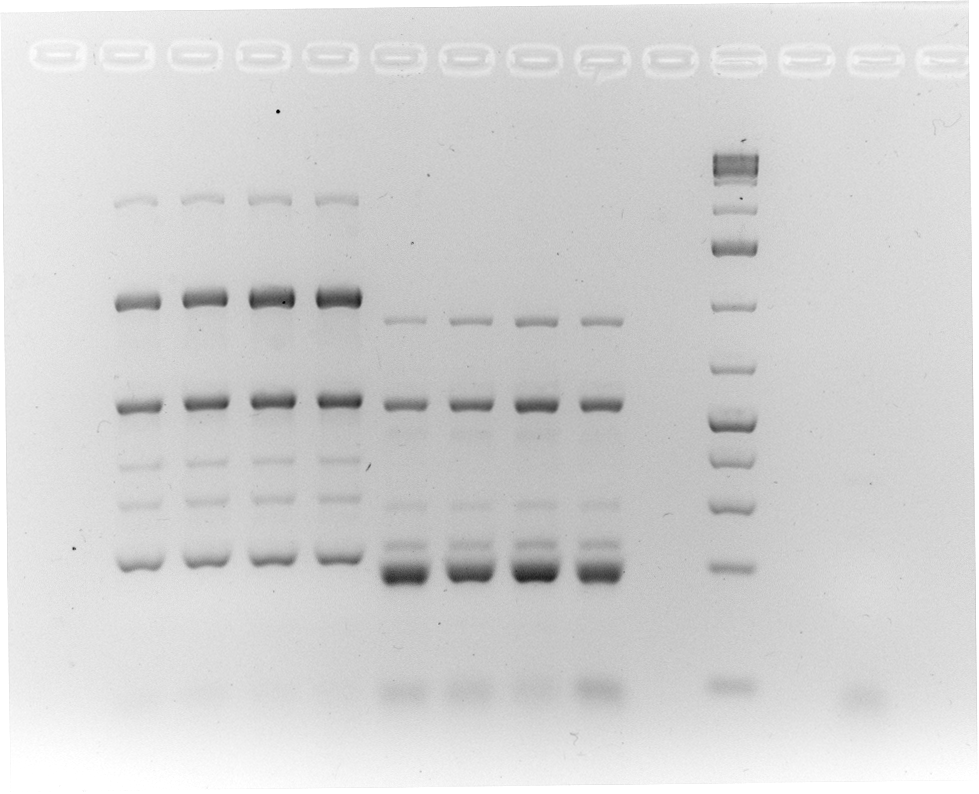

Supplement: S1 Fig — (TIF) [file pone.0237111.s004.tif]
