# Supplementary material for: aTBP: A versatile tool for fish genotyping
Source: PLoS One. 2020 Aug 4;15(8):e0237111. doi: 10.1371/journal.pone.0237111 (PMC7402489; doi:10.1371/journal.pone.0237111)
Supplement: Attachment — Submitted filename: comments -plos04.docx [file pone.0237111.s005.docx]

The manuscript by Gianì et al. entitled “aTBP: a versatile tool for fish genotyping”, the author developed new genotyping method and applied to the identification of fish species. This article develops the new method to indicate the relationship of inter- and intra- species. But, I found there are some mistakes as well as experimental method in this manuscript. The author only uses seven fish species for this research and these samples come from the market. Farmed individuals may come from a single ancestor. The sample is too small and not representative. The author must use more species and evidence to prove that this method is useful. Teleost fishes represent a highly diverse group consisting of more than 20,000 species. The author cannot prove that this method can distinguish all species. The author must substantially modify the method and the description of the results. I consider the methodology of this article to be inappropriate in their current form, in my opinion, this manuscript does not meet criteria for publication and must therefore be reject.
