# Supplementary material for: aTBP: A versatile tool for fish genotyping
Source: PLoS One. 2020 Aug 4;15(8):e0237111. doi: 10.1371/journal.pone.0237111 (PMC7402489; doi:10.1371/journal.pone.0237111)
Supplement: Attachment — Submitted filename: Rebuttal letter_RESPONSE TO REWIEVERS_DBfinal.docx [file pone.0237111.s006.docx]

**PONE-D-20-08308
aTBP: a versatile tool for fish genotyping
PLOS ONE**

**RESPONSE TO REWIEVERS**

**Q**: *To enhance the reproducibility of your results, we recommend that if applicable you deposit your laboratory protocols in protocols.io, where a protocol can be assigned its own identifier (DOI) such that it can be cited independently in the future.*
**A**: Concerning your kind suggestion about the deposit of our laboratory protocol please note that we have already detailed it in a recent publication (Gianì et al.,Food Control, 2019, <https://doi.org/10.1016/j.foodcont.2019.107010> Online 19Nov2019) when reporting for the first time the development and the use of the aTBP method. Thanks for the offer.

**EDITING**

**Q**: *PLOS ONE now requires that authors provide the original uncropped and unadjusted images underlying all blot or gel results reported in a submission’s figures or Supporting Information files*
**A**: As requested, the original uncropped image of the agarose gel of Fig. 2 is now submitted as supporting information (S4).

**Q** : *Please provide an amended statement that declares *all* the funding or sources of support (whether external or internal to your organization) received during this study, as detailed online in our guide for authors at* [*http://journals.plos.org/plosone/s/submit-now*](http://journals.plos.org/plosone/s/submit-now)*.  Please also include the statement “There was no additional external funding received for this study.” in your updated Funding Statement.*
**A** : As requested, we specify that : There was no additional external funding received for this study and the funders had no role in study design, data collection and analysis, decision to publish, or preparation of the manuscript.”

**Q** : *We note that you have included the phrase “data not shown” in your manuscript. Unfortunately, this does not meet our data sharing requirements. PLOS does not permit references to inaccessible data. We require that authors provide all relevant data within the paper, Supporting Information files, or in an acceptable, public repository. Please add a citation to support this phrase or upload the data that corresponds with these findings to a stable repository (such as Figshare or Dryad) and provide and URLs, DOIs, or accession numbers that may be used to access these data. Or, if the data are not a core part of the research being presented in your study, we ask that you remove the phrase that refers to these data.*
**A**: As requested, we are now providing in a text format, derived from FASTA format, a supporting information file reporting all the COI sequences, in both aligned and unaligned frame, of the fish samples bought at the market (S3 list). As reported in the M&M section, they result from the sequencing and cross matching of both strands

**REF 1
Q** : *The manuscript by Gianì et al. entitled “aTBP: a versatile tool for fish genotyping”, the author developed new genotyping method and applied to the identification of fish species. This article develops the new method to indicate the relationship of inter- and intra- species. But, I found there are some mistakes as well as experimental method in this manuscript. The author only uses seven fish species for this research and these samples come from the market. Farmed individuals may come from a single ancestor. The sample is too small and not representative. The author must use more species and evidence to prove that this method is useful. Teleost fishes represent a highly diverse group consisting of more than 20,000 species. The author cannot prove that this method can distinguish all species. The author must substantially modify the method and the description of the results. I consider the methodology of this article to be inappropriate in their current form, in my opinion, this manuscript does not meet criteria for publication and must therefore be reject.*
**A:** We feel sorry to realise that our contribution has not found the appreciation of referee n.1. We are afraid that he/she could have possibly overlooked or missed some of the delivered information. With reference to the sampling, Ref.1 seems to have failed to appreciate that the vast majority of the analyzed fish samples came from aquaculture and were preliminary characterized at both morphological and at molecular levels with a panel of SSRs. We have clearly stated this in the M&M section and all over the manuscript, providing references to related projects and publications. This material was purposely and properly used to verify the reliability of our aTBP findings, acting as a gold standard. In addition we have collected samples from a completely different source, that is the fish market, authenticated with the COI marker so to build up a 3 markers crossed/referenced data : SSRs, COI and aTBP, an experimental strategy that serves the purpose of validating the aTBP method. The number of the analyzed species , seven, was limited by the availability of enough individuals and data that could corroborate the 3-markers approach and yet major species of commercial and scientific interests (tuna, sturgeon, gilthead seabream, European seabass, salmonids) have been included. Inclusion of additional fish species would have been redundant not adding more information, and would have made the paper too long and burdensome. With reference to the number of species distinguishable by aTBP, we certainly cannot exclude that out of 20.000 teleost species some aTBP profiles could overlap but that, in our opinion, would also be informative about their close genetic relationships very much similar to what we have shown for S. carpio, postulated as emerging from S. trutta fario as a recent speciation event. The common ancestor argument raised, is very much theoretical and in principle we cannot drop it but the experimental evidences we have collected from two different groups of sampling, fish market and aquaculture, where some intraspecific DNA polymorphisms are shared, tend to exclude it. Anyhow the aTBP tool applied to fish genotyping is also addressing the future, that is the monitoring of population changes that are in progress or will be in response to several adaptation events, thus providing a method that is very convenient , sustainable and affordable for many laboratories.

**REF 2**
**Q**: *Extra editing is necessary.*
**A** : It has been done in accordance to PLOS ONE style

**Q**. *The authors should discuss about how to deal with polyploid species, which would bring many more variants to improve the practical difficulty in genotyping. By the way, it would be much better if a practical database can be established. These issues should be mentioned in the discussion section.*
**A**: We thank the referee for his/her kind consideration of our work. With reference to ploidy, as it has been documented for sturgeon and the salmonids, known polyploid species with a prevalent 4N and 8N evolutionary/natural scale ploidy (4N functional scale) and very different chromosome numbers (from 54/58 to 240/264), aTBP is well performing and we think that its discrimination capacity could extend even further to a higher ploidy level since the major restriction of the technique is the limit of-resolution of the amplified fragments in CE, that is 1-2 bp. Clearly a higher number of target sequences would increase the number of peaks, not linearly because it would depend from the allelic variance, and this call for a faster reading, recognition and comparison of the profiles. This is the reason we are currently developing a software that could efficiently y compare the aTBP profile of the analyzed samples with authentic profiles of reference. This also calls for the establishment of a dedicated data base, as correctly suggested by the referee. We are doing this within the limit of our possibility because that requires a full collaboration with Institutions that can provide certified material . We are also in contact with some producers (caviar/ sturgeon) to expand our data. As requested by the referee we report these considerations in the discussion section together with a couple of new references.
