# Supplementary material for: aTBP: A versatile tool for fish genotyping
Source: PLoS One. 2020 Aug 4;15(8):e0237111. doi: 10.1371/journal.pone.0237111 (PMC7402489; doi:10.1371/journal.pone.0237111)
Supplement: Attachment — Submitted filename: Comment-0613.docx [file pone.0237111.s007.docx]

In this study, the authors initially found size polymorphism in aTBP fragments, and examined this scenario further with evidence in other 7 fish species. The authors concluded the statement below, by the discovery of varying size fragments existing within and between species as well as at inter-population level,

“*These data are discussed with respect to the application of the aTBP method to diverse fields of investigation that may include the characterization of a fish population and assessment of its variations in response to environmental changes, the recognition of genetic diversification resulting from hybridization events and studies on parental assignment as well as species traceability, authentication and detection in seafood.*”

The above derivation, regarding to the function of aTBP, seems to be a courageous assumption while many unknows are yet, or left to be answered.

However, aTBP is a gene locus residing on the certain region of microsatellite, and the scoped and resolution in population genetic study would be constrained by analyzing various fragment sizes of homologous alleles of single gene, especially limited information revealed by fragment sizes only. Simply speaking, it is rarely, or nearly practical to study population genetic on targeted species through single genetic locus of one single microsatellite gene as found in this study.

Otherwise, fragment size is not recommended studying population genetic structure for its unknown mutation mode or mechanism affecting fragment length difference. For example, a set of homologous genes with similar genetic length may probably have difference nucleotide composition, in which a wrong conclusion might be easily obtained under this assumption.

By thoroughly consideration, despite the significant efforts from the authors, this paper of describing genotyping methodology to fish may present limited value and depth, as not claimed by the authors. Regretfully, I would suggest rejecting this paper as my final decision.
