# Supplementary material for: aTBP: A versatile tool for fish genotyping
Source: PLoS One. 2020 Aug 4;15(8):e0237111. doi: 10.1371/journal.pone.0237111 (PMC7402489; doi:10.1371/journal.pone.0237111)
Supplement: Attachment — Submitted filename: REV2 REBUTTAL LETTER_DB.docx [file pone.0237111.s008.docx]

**REBUTTAL LETTER TO ACADEMIC EDITOR AND TO REFEREE COMMENTS**.
Dear Editor, please find the reply to the criticisms moved by the referees

REF1 Remarks : This time ref1 objection, different from those made on the first version of the manuscript, seems to be motivated by his/her reluctancy to accept that aTBP can be a useful tool for studying popolulation genetics that is allele variance that may occur in a given popolutation across time and in response to different external changes. We say it seems because his/her consideration, as far as we can tell from his/her wording, starts from an ill-based assumption that is that aTBP is .. a gene locus residing on the certain region of microsatellite (cited) .. . As reported TBP is instead based on intron-length variation occurring in the numerous and different members of the beta-tubulin gene family. Thus, it is not a single locus marker neither a microsatellite sequence, not even … one single microsatellite gene (cited) . We are afraid that this misconception, although limited to a possible application of otherwise unquestioned experimental data, can lead to erroneous conclusions. Nevertheless, since it looks that ref 1 didn’t like the sentence of the abstract referring to the different applications of aTBP, with specific focus on population genetics , we have changed it to please him/her and make it even more fitting to the experimental data.

REF2 remarks : With respect to ref 2 criticism about poor attention to the overall writing of the manuscript …and his/her recommendation for English editing .., please note that the text have been revised by Prof. Khidir Hilu of Virginia Tech, USA and Prof. Sara Patterson, Emeritus at Wisconsin University USA.
